# Assessment of a revolving drug fund for essential asthma medicines in Benin

**DOI:** 10.1186/s40545-015-0033-7

**Published:** 2015-04-13

**Authors:** Gildas Agodokpessi, Nadia Aït-Khaled, Martin Gninafon, Leon Tawo, Wilfried Bekou, Christophe Perrin, Karen Bissell, Nils Billo, Donald A Enarson, Chen-Yuan Chiang

**Affiliations:** Centre National Hospitalier de Pneumo phtisiolologie, Cotonou, Bénin; International Union Against Tuberculosis and Lung Disease, 68, boulevard Saint-Michel, 75006 Paris, France; Division of Pulmonary Medicine, Department of Internal Medicine, Wan Fang Hospital, Taipei Medical University, Taipei, Taiwan; Department of Internal Medicine, School of Medicine, College of Medicine, Taipei Medical University, Taipei, Taiwan

**Keywords:** Asthma, Revolving drug fund, Beclometasone, Inhaled corticosteroid

## Abstract

**Objectives:**

Benin established a revolving drug fund (RDF) for essential asthma medicines in 2008. We evaluated the operation of the RDF and assessed whether there was interruption of supply of asthma medicine from 2008 to 2013.

**Methods:**

We reviewed the process in establishing the RDF. We assessed cost and sale price of asthma medicines, expenditure of the RDF in procuring asthma medicines and other tools, revenue generated by sales of medicines to patients, and balance of capital as of 31 January 2013. We investigated whether there was interruption of supply of essential asthma medicines from 2008–2013.

**Results:**

The total amount of grants initially injected into the RDF was 24,101€. As of 31 January 2013, the capital of the RDF, including the deposit in the RDF bank account (8,114€) and the value of inhalers in stock (12,172€), was equivalent to 20,586€, slightly less than the initial capital (24,101€). The decrease of capital was mainly because a number of inhalers were expired or provided free-of-charge (6,091€) and because part of the fund was used to procure other elements required for the management of asthma (4,338€). Thanks to a RDF, Benin maintained an uninterrupted supply of essential asthma medicines in asthma pilot sites from 2008–2013.

**Conclusion:**

The Benin experience demonstrated that in countries where universal health coverage was not yet in place, establishment of a RDF may help maintain an uninterrupted supply of essential medicines.

## Introduction

Shortage of pharmaceutical supply is common in resource-limited settings. Access to affordable essential medicines for non-communicable diseases in resource limited settings can be particularly challenging [1,2]. The low affordability of essential asthma medicines, in particular the inhaled corticosteroid (ICS), was identified as one of the main barriers in providing proper asthma care in low- and middle-income countries [2-7]. Revolving drug funds have been applied in a variety of settings to address this problem through financing drug supply [8-11]. It usually begins with an initial capital investment, followed by replenishment of drug stock with monies collected from the sale of medicines.

A project introducing a comprehensive approach to lung health (CAL), funded by the World Bank and implemented through collaboration between the International Union Against Tuberculosis and Lung Disease (The Union) and partners in Benin, China and Sudan, included as one of its aims to reduce the burden of lung disease through management of patients with persistent asthma [12,13]. In Benin, it was implemented in 2008 at two public health facilities (Centre national hospitalier de pneumophtisiologie (CNHP) in Cotonou and one tuberculosis unit located in Porto-Novo) and three missionary health facilities. For the implementation of standard case management of asthma, it was decided to establish a revolving drug fund (RDF) for essential asthma medicines to ensure uninterrupted supply of essential asthma medicine. In this paper, we evaluated the operation of a RDF for asthma from 2008 to 2013.

## Methods

### Setting

Benin is a low income country where universal health coverage is not yet in place. Most asthma patients have had to pay out of pocket for health services. Before the asthma pilot project, the majority of asthma patients were being treated exclusively on an emergency basis when they presented with an acute attack of asthma and that only a small minority of asthma patients were prescribed with ICS in Benin [12].

### Establishment of a revolving drug fund for essential asthma medicine

Before the CAL project implementation, the Benin national tuberculosis programme (NTP) manager and The Union consultant discussed whether asthma inhalers should be provided free-of-charge to asthma patients or whether it would be more sustainable to establish a RDF for essential asthma medicines. As asthma is a chronic disease and long term management is required, maintaining uninterrupted supply of affordable asthma medicines is essential for ensuring the continuity of care. There was concern that if asthma inhalers were provided at no cost to patients, the supply of asthma inhalers might be interrupted when the project finished. Thus, part of the World Bank grant was used as an initial capital investment to establish a RDF for asthma medicines that would aim to sustain long term management of asthma in five asthma pilot sites.

The coordinator of the asthma projects and the RDF was the NTP manager, who was also the director of the biggest site for asthma management (the CNHP). A dedicated bank account for the RFD was created; monies collected from the sale of asthma inhalers were deposited in the RFD bank account for further procurement of asthma inhalers.

### Initial capital of RDF

The initial capital invested into the CAL project in 2008 for procuring asthma medicines was 9,940€. With this, Benin’s NTP manager purchased 2,000 units each of inhaled beclometasone (200 puffs, 250 μg per puff) and of inhaled salbutamol through Benin’s central pharmacy for essential medicines (CAME). Due to a delay in delivery of medicines at the beginning of project implementation, the CAME donated 320 inhalers of beclometasone (total 1,203€), which allowed the project to commence and added to the value of the RDF. Until the ordered medicines arrived, patients had to buy inhaled salbutamol at a private pharmacy. The total grant provided to the CNHP to establish the RDF for asthma for the CAL project, including the medicines donated by the CAME, was 11,143€.

### Asthma drug facility and additional capital of RDF

The Union created the Asthma Drug Facility (ADF), which aimed to provide affordable access to quality-assured essential asthma medicines for low- and middle-income countries [14,15]. The ADF pooled requests from qualifying programmes for quality-assured essential asthma medicines. It used bi-yearly international competitive bids to offer the most affordable prices possible for asthma medicines after a qualification procedure ensuring the selection of inhalers in strict compliance with international quality standards. To pilot the operations of the ADF, The Union and the Benin NTP agreed that The Union would donate inhaled medicines and provide training and technical assistance for the implementation of standard case management of asthma, and that the Benin NTP would maintain the RDF for asthma to procure essential asthma medicines through the ADF for the following three years. It was agreed that a small margin should be added to the cost of the medicines procured through the ADF to cover customs clearance, storage, distribution, and potential loss of drugs, and to slightly increase the amount of the fund to ensure sustainability. The Union donated 2,500 units of inhaled beclometasone propelled by hydrofluoroalkanes (HFA) (100 μg per puff, 200 puffs) and 2000 HFA inhaled salbutamol (100 μg per puff, 200 puffs), equivalent to 7530€, to Benin through the ADF in 2010 for the implementation of the ADF pilot project.

In addition to medicines, both the CAL project and the ADF pilot project funded other project elements, such as training, coordination, supervision, purchase of peak flow meter, spirometers, and mouthpieces, and the printing of asthma treatment cards and treatment registers as well as posters for patient education. At the end of the projects, 5,428€ remained unspent and were injected into the RDF.

### Maintaining the RDF

The CNHP was responsible for the distribution of inhalers to other pilot sites and maintaining the RDF. The sites charged patients at the selling price recommended by CNHP without adding a site margin during the first project year. All these sites agreed to conduct quarterly follow-up and to manage asthma attacks free-of-charge (the usual consultation fee was about 0.30€). After the end of the one year ADF pilot project, the three missionary sites decided unilaterally to add a site margin for the management of asthma drugs and increased the cost of both inhaled beclometasone and inhaled salbutamol by 0.3€ per inhaler, which did not return to the RDF. In March 2011, the Benin NTP Director, after discussion with The Union consultants, decided to impose a markup of 0.30€ per inhaler as a site margin in order to standardise the selling price of inhalers and to maintain the services of follow-up at no cost to patients at all sites.

### Medicine management tool

A medicine management tool using Microsoft Excel has been developed for the management of asthma inhalers. It was used periodically to assess number of inhalers in stock at the beginning of a period; number of inhalers ordered during that period; number of inhalers in stock at the end of the period; number of inhalers used during the period; average monthly consumption of inhalers; number of months that inhalers in stock can cover. This information was then used to estimate the date for placing the next order of inhalers, aiming to order 8 months before the inhalers would run out (a two-month buffer plus six months as the estimated interval between placing an order to receiving the medicines).

### Evaluation of the RDF

To evaluate the RDF in Benin, we reviewed the costs of procuring the asthma medicines, the price at which medicines were sold to patients, costs for procuring other elements related to the management of asthma (peak flow meters, mouthpieces, printing of asthma treatment cards and registers), the revenue generated by selling medicines to patients, and the balance of capital as of 31 January 2013. We assessed the performance of the medicine management tool and check whether there was interriuption of essential asthma medicine at projct sites.

The asthma projects focused on long-term management of patients with persistent asthma. The second edition [16] of The Union’s guide for standard case management of asthma was used in the CAL project; the third edition [17] was used in the ADF project. We reviewed registration of asthma patients for standard case management of asthma during the period 2008–2011.

### Ethics

As the information collected was a part of routine health service operations in Benin, review by an ethics committee was not considered to be required. All information was handled by the health care workers who provided care for the patients on a routine basis. No individual identifiers were provided to individuals outside the health service.

## Results

Table 1 shows that the total amount of grants injected into the RDF over the period of 2008–2010 was 24,101€.

**Table 1:** **Grants that contributed to Benin’s revolving drug fund, 2008–2013**

	**Number of inhalers**	**Price per inhaler (€)**	**Amount of money (€)**
1. CAL Project			11 143
Grant from World Bank			
Inhaled beclometasone	2,000	3.76	7,520
Inhaled salbutamol	2,000	1.21	2,420
Donation from CAME			
Inhaled beclometasone	320	3.76	1,203
2. Donation from Asthma Drug Facility project			7,530
Inhaled beclometasone	2,500	1.78	4,450
Inhaled salbutamol	2,000	1.54	3,080
3. Grant in medicines (1 + 2)			18,673
4. Grant in cash			5,428
5. Total revolving fund grant (3 + 4)			24,101

Note: CAL, comprehensive approach to lung health project; CAME, central pharmacy for essential medicines of Benin.

### Medicines prices for the CAL and ADF projects

Table 2 shows that in the CAL project the purchase price of inhaled salbutamol, propelled by chlorofluorocarbons (CFC), was 0.95€ and that of CFC inhaled beclometasone 3.50€. With a 14% margin set by the NTP, the selling price was 1.37€ for inhaled salbutamol and 4.27€ for inhaled beclometasone.

**Table 2:** **Purchase price, freight fee, margin and selling price for inhaled beclometasone and inhaled salbutamol in a comprehensive approach to lung health project and an asthma drug facility pilot project in Benin**

	**Inhaled Beclometasone (€)**	**Inhaled Salbutamol (€)**
CAL Project		
a) Purchase price	3.50	0.95
b) Freight and other fees	0.26	0.26
c) Cost of drug (a + b)	3.76	1.21
d) Margins (about 14%)	0.51	0.16
e) Selling price (c + d)	4.27	1.37
ADF Project		
f) Purchase price	1.07	0.83
g) Freight and other fees	0.49	0.49
h) Transit fee	0.22	0.22
i) Cost of drug (f + g + h)	1.78	1.54
j) Margin (about 10%)	0.2	0.13
k) Selling price (i + j)	1.98	1.68

Note: CAL, comprehensive approach to lung health project; CAME, central pharmacy for essential medicines of Benin.

The cost of ADF inhaled beclometasone was 1.78€, about half of the cost of inhaled beclometasone procured locally during the CAL project. The cost of ADF inhaled salbutamol was 1.54€, about 27% higher than that of inhaled salbutamol procured locally during the CAL project (Table 2). With an average of 10% margin, the selling price was 1.98€ for inhaled beclometasone and 1.68€ for inhaled salbutamol in the ADF pilot project.

### Expenditure and recovery of the Revolving Drug Fund as of January 2013

Between 2008 to 2013, the Benin NTP placed three orders for a total of 7,386 inhaled beclometasone and four orders for a total of 8,865 inhaled salbutamol with an expenditure of 24,887€ through the ADF. In addition, the Benin NTP spent 4,338€ procuring peak flow meters and mouthpieces through the ADF, and printing asthma posters, asthma treatment cards and registers. The total expenditure of the RDF was 29,225€ (Table 3).

**Table 3:** **Asthma revolving drug fund expenditure for medicines and other items related to asthma management, Benin 2008–2013**

**Orders**	**Number of inhalers procured (1)**	**Purchase price per inhaler (€) (2)**	Purchase price, total inhalers (€) (3 = 1*2)	**Transport and ADF fees (€) (4)**	**Total cost (€) (3 + 4)**
Beclometasone					
1st order	1,386	1.07	1,483	2,557	4,040
2nd order	3,000	0.99	2,970	1,135	4,111
3rd order	3,000	0.99	2,970	2,747	5,717
(a) Subtotal	7,386		7,423	6,438	13,867
Salbutamol					
1st order	681	0,83	565	329	894
2nd order	3,114	0.83	2,585		2,585
3rd order	2,520	0.84	2,117	1,178	3,287
4th order	2,550	0.83	2,117	2,149	4,254
(b) Subtotal	8,865		7,382	1,507	11,020
(c) Expenditure for procuring inhalers (a + b)					24,887
(d) Other expenditure					4,338
Total expenditure (c + d)					29,225

Table 4 shows the number of inhalers to be sold and selling price per inhaler. Complete collection of money from the sale of these inhalers would generate 50,474€ for the RDF. Table 5 shows that a total of 32,211€ has been recovered so far from selling inhalers to patients. The value of the inhalers in stock was equivalent to 12,172€. The value of inhalers that were expired or provided to the poorest patients free-of-charge amounted to 6,091€.

**Table 4:** **Number of inhalers to be sold, selling price per inhaler and money to be recovered for asthma revolving drug fund in Benin by 31 January 2013**

**Medicines**	**Number of inhalers**	**Sellling price per inhaler (€)**	**Money to be recovered by selling inhalers (€)**
CAL project			
Beclometasone	2,320	4.27	9,906
Salbutamol	2,000	1.37	2,740
ADF project			
Beclometasone	9,886*	1.98	19,574
Salbutamol	10,865†	1.68	18,253
Total			50,474

Note: CAL, comprehensive approach to lung health project; ADF, Asthma Drug Facility.

*Includes 2500 donated by the ADF and 7386 procured through the ADF.

†Includes 2000 donated by the ADF and 8865 procured through the ADF.

**Table 5:** **Funds recovered by the asthma revolving drug fund in Benin by 31 January 2013**

**Medicines**	**Number of inhalers**	**Selling price per inhaler (€)**	**Value of inhalers (€)**
1) Lost or provided free			6,091
CAL project			
Beclometasone	879	4.27	3,753
Salbutamol	250	1.37	343
ADF project			
Beclometasone	283	1.98	560
Salbutamol	854	1.68	1,435
2) Money already recovered			32,211
CAL project			
Beclometasone	1,441	4.27	6,153
Salbutamol	1,750	1.37	2,398
ADF project			
Beclometasone	6,213	1.98	12,302
Salbutamol	6,761	1.68	11,358
3) ADF inhalers in stock			12,172
Beclometasone	3,390	1.98	6,712
Salbutamol	3,250	1.68	5,460
Total (1 + 2 + 3)			50,474

Note: CAL, comprehensive approach to lung health project; ADF, Asthma Drug Facility.

Table 6 shows that the capital of the RDF as of 31 January 2013, including the deposit in the RDF bank account (8,114€) and the value of inhalers in stock (12,172€), was equivalent to 20,586€, which was less than 24,101€, the total amount of capital initially injected into the RDF. The decrease in the RDF was mainly because a number of inhalers were expired or provided free-of-charge (6,091€) and because part of the fund was used to procure other items required for the management of asthma patients (4,338€).

**Table 6:** **Capital of asthma revolving drug fund in Benin, as of January 2013**

	**Total amount (€)**
1. Balance in Bank (a + b-c)	8,414
a) Grant in cash	5,428
b) Monies recovered from medicines	32,211*
c) Expenditure	29,225†
2. Value of inhalers in stock	12,172‡
Total (1 + 2)	20,586

*See Table 5, money already recovered.

†See Table 3, total expenditure.

‡See Table 5, ADF inhalers in stock.

### Estimations for the next order

Table 7 shows the use of the tool on 31 January 2013, which estimated that the date for placing the next order of inhalers would be in July 2013. Consequently, the NTP Benin sent an order to ADF in July 2013 to prevent interruption of supply.

**Table 7:** **Estimation of the date for placing the next order of asthma inhalers, revolving drug fund, Benin, 31 January 2013**

**Medicines**	**Number of inhaler in stock (a)**	**Date begin (b)**	**Medicines ordered during the period (c)**	**Date end (d)**	**Number of inhalers remaining (e)**	**Consumption during the period (f = a + c-e)**	**Number of months during the period (g)**	**Average monthly consumption (h = f/g)**	**Estimation of the number of months covered by inhalers in stock (i = f/h)**	Approximate date of placing next medicine order (i-8*) months since (d)
Beclometasone	1 600	15/09/2012	3 000	31/01/2013	3 390	1 210	4.6	263	12,9	26/06/2013
Salbutamol	2 130	15/09/2012	2 250	31/01/2013	3 250	1 130	4.6	246	13,2	06/07/2013

*Ordering must be started as soon as the number of inhalers in stock is sufficient for only 8 months: 2 months for reserve stock and 6 months as turnaround time between ordering and arrival of inhalers.

The assessment found that there was no interruption of supply of asthma medicines from 2008–2013 in asthma pilot sites in Benin.

### Asthma patient management

The results of asthma management during the CAL project have been reported [11]. Since the beginning of the ADF pilot project in April 2010, asthma management has been performed consistently and quarterly reports sent to The Union during the three years of the project. The number of patients with persistent asthma newly registered was 204 in the first project year (April 2010-March 2011). It increased to 231 in the second year (April 2011-March 2012) and to 274 in the third year (April 2012-March 2013). In addition to the 709 newly registered cases with persistent asthma, patients with intermittent asthma and patients registered prior to the ADF pilot project were also treated with medicines provided through the ADF.

## Discussion

Before the asthma pilot project, only a small minority of asthma patients were prescribed with ICS in Benin [12]. The CAL asthma pilot project began with national adaption of international asthma guidelines, followed by training of clinicians for guideline implementation. To ensure sustainability of the long-term management of asthma, an uninterrupted supply of ICS is essential, for which a RDF of asthma was established in Benin. The RDF provided ICS at affordable prices for the majority of asthma patients. For a small minority of poor asthma patients who could not afford to pay, the ICS were provided at no cost.

Since the beginning of the asthma project in 2008, long-term management of asthma using ICS has reduced suffering and improved the quality of life of asthma patients. The proportion of asthma patients who had at least one visit to emergency services in the previous year for an asthma attack was 59.4% before the project, which reduced to 1.6% after the project [12].

Since the establishment of the RDF, the capital of the fund was always sufficient to ensure uninterrupted supply of asthma medicines in the five pilot sites. Management of patients with persistent asthma using ICS has became routine practice in the pilot sites. The tool for managing medicine ordering was useful as it estimated the number of months covered by inhalers in stock and the approximate date when the next medicine order should be placed. The tool did not use the number of asthma patients to estimate consumption because the severity of asthma may vary over time and patients may not follow advice to continue treatment when their asthma is under control. If the RDF is maintained properly, it might be possible to extend the management of asthma to other sites using medicines provided by the RDF.

Several challenges should be highlighted. First, the government’s political commitment for national asthma control was not yet in place and funding for the management of non-communicable diseases was lacking. It is challenging to ensure sustainibility of the long-term management of asthma. Second, maintaining a private bank account for the RDF in a public health structure and keeping the health personnel motivated to manage the distribution of medicines and the replenishment of stock with monies collected from the sale of medicines over a prolonged period of time might be challenging. Several issues arose during project implementation, such as expiry of medicines, money from medicine sales not recovered, medicines provided for free but not recorded, and money from medicine sales recovered but not registered. Consequently, the capital of the RDF was not fully recovered, resulting in a decrease of capital after five years of operation.

The Pan American Health Organization (PAHO) has established the PAHO Strategic Fund to assist PAHO member states in the procurement of essential medicines and basic public health products [18]. In 2013, PAHO started to add medicines for Non-Communicable Diseases (NCD) to this fund. However, there is no such regional procurement mechanism in place in Africa. The World Health Organization (WHO) has been advocating for universal health coverage to ensure access to health care, for which countries must raise sufficient funds, reduce the reliance on direct payments to finance services, and improve efficiency and equity [19]. Furthermore, the WHO Global Action Plan for Prevention and Control of NCD 2013–2020 has set the target of “80% availability of the affordable basic technologies and essential medicines” [20]. This target applies to asthma inhalers. Until universal health coverage is implemented and providing access to care for asthma patient in Benin, the RDF will continue playing an important role in ensuring an uninterrupted supply of essential asthma medicines.

## Conclusion

The Benin experience demonstrated that in countries where universal health coverage was not yet in place, establishment of a RDF may help maintain an uninterrupted supply of essential medicines.

## Abbreviations

ADF: Asthma Drug Facility
CAL: Comprehensive approach to lung health
CAME: Central pharmacy for essential medicines
CFC: Chlorofluorocarbons
CNHP: Centre national hospitalier de pneumophtisiologie
ICS: Inhaled corticosteroid
HFA: Hydrofluoroalkanes
NCD: Non-Communicable Diseases
NTP: National tuberculosis programme
PAHO: Pan American Health Organization
RDF: Revolving drug fund
The Union: International Union Against Tuberculosis and Lung Disease
WHO: World Health Organization
